# Experimental *Piscine orthoreovirus* infection mediates protection against pancreas disease in Atlantic salmon (*Salmo salar*)

**DOI:** 10.1186/s13567-016-0389-y

**Published:** 2016-10-21

**Authors:** Morten Lund, Magnus Vikan Røsæg, Aleksei Krasnov, Gerrit Timmerhaus, Ingvild Berg Nyman, Vidar Aspehaug, Espen Rimstad, Maria Krudtaa Dahle

**Affiliations:** 1 Section of Immunology, Norwegian Veterinary Institute, Oslo, Norway; 2 SalMar ASA, Kverva, Norway; 3 Department of Food Safety and Infection Biology, Norwegian University of Life Sciences, Oslo, Norway; 4 Nofima AS, Norwegian Institute of Food, Fisheries and Aquaculture Research, Ås, Norway; 5 PatoGen AS, Ålesund, Norway

## Abstract

Viral diseases are among the main challenges in farming of Atlantic salmon (*Salmo salar*). The most prevalent viral diseases in Norwegian salmon aquaculture are heart and skeletal muscle inflammation (HSMI) caused by *Piscine orthoreovirus* (PRV), and pancreas disease (PD) caused by Salmonid alphavirus (SAV). Both PRV and SAV target heart and skeletal muscles, but SAV additionally targets exocrine pancreas. PRV and SAV are often present in the same locations and co-infections occur, but the effect of this crosstalk on disease development has not been investigated. In the present experiment, the effect of a primary PRV infection on subsequent SAV infection was studied. Atlantic salmon were infected with PRV by cohabitation, followed by addition of SAV shedder fish 4 or 10 weeks after the initial PRV infection. Histopathological evaluation, monitoring of viral RNA levels and host gene expression analysis were used to assess disease development. Significant reduction of SAV RNA levels and of PD specific histopathological changes were observed in the co-infected groups compared to fish infected by SAV only. A strong correlation was found between histopathological development and expression of disease related genes in heart. In conclusion, experimentally PRV infected salmon are less susceptible to secondary SAV infection and development of PD.

**Electronic supplementary material:**

The online version of this article (doi:10.1186/s13567-016-0389-y) contains supplementary material, which is available to authorized users.

## Introduction

Virus infections are a continuous challenge in large-scale aquaculture of Atlantic salmon (*Salmo salar*). Environmental factors, high intensity production and infectious agents affect both welfare and production [1–3]. The two most prevalent viral diseases in Norwegian Atlantic salmon aquaculture are heart and skeletal muscle inflammation (HSMI) and pancreas disease (PD) [4]. *Piscine orthoreovirus* (PRV) is associated with HSMI, is ubiquitous in sea reared Atlantic salmon in Norway and often detected without any signs of disease [5, 6]. Pancreas disease is caused by Salmon pancreas disease virus, more commonly known as Salmonid alphavirus (SAV). The two viral diseases have overlapping geographic distributions [4, 7], both target heart and skeletal muscle and may co-infect Atlantic salmon [8–10].

PRV is a non-enveloped virus with a segmented, double stranded RNA genome, belonging to the genus *Orthoreovirus* in the family *Reoviridae* [5, 11]. Salmonid erythrocytes are major target cells for PRV and more than 50% of these cells may be infected in the peak phase of the infection [12]. In later stages of the infection, PRV infects myocytes of the heart and skeletal muscles [13]. The histopathological changes in heart and skeletal muscle gave the condition its name in the late 1990s, and later the association with PRV was established [5, 14].

SAV is an enveloped virus with a single-stranded positive sense RNA genome of the family *Togaviridae* [15]. Pancreas, heart and skeletal muscle are the main target tissues. The disease is recognized by growth retardation, reduced slaughter quality and increased mortality [9, 16, 17]. Histopathological changes are characterized by acute necrosis of exocrine pancreas, myocardial and skeletal muscle necrosis with subsequent inflammation [9]. The pancreatic lesions are a hallmark of PD and hence used for diagnostic differentiation from HSMI and cardiomyopathy syndrome (CMS) [9]. However, PRV and SAV have common target tissues in heart and skeletal muscles, making interactions between the two viral infections possible.

SAV is divided into six different phylogenetic subtypes [18] and subtypes 2 and 3 are present in Norwegian aquaculture [19, 20]. The two subtypes show approximately 7% differences in nucleotide sequence [19], are endemically present in separate geographic areas and differ in virulence [20–22]. The mechanisms behind the difference in virulence are unknown. No stereotypical difference is described between subtype 2 and 3 [23]. PD outbreaks vary in duration, severity and accumulated mortality [24], indicating that factors other than SAV influences disease development. Interaction with other infectious agents may be such a factor.

Protection to a secondary virus infection induced by an unrelated primary virus infection has been recognized since the 1950s [25], and has also been described for several viruses infecting salmonid fish [26–30]. However, the duration of the protection of rainbow trout to infectious hematopoietic necrosis after primary infection with the non-virulent cutthroat trout virus was found to be no more than 4 weeks [26]. In addition, some viral infections in terrestrial animals are shown to aggravate disease development of a secondary viral infection [31, 32].

The purpose of this study was to determine if a primary PRV infection alters the outcome of a subsequent SAV infection. Experimental infection trials were performed to compare disease development, viral kinetics and expression of disease associated genes between PRV-SAV co-infected and SAV infected fish.

## Materials and methods

### Fish

Sea water adapted Atlantic salmon (*N* = 987) of a SalmoBreed strain (Bergen, Norway) were used in the study (VESO Vikan, Namsos, Norway). The post smolts were transferred to sea water two weeks before PRV challenge. Prior to challenge, the fish were screened and found to be negative for PRV, infectious pancreatic necrosis virus (IPNV) and SAV by reverse transcriptase (RT) qPCR. PRV shedders (*N* = 5) sampled four weeks after PRV challenge were confirmed negative for Atlantic salmon calicivirus [33]. During the challenge trial the fish were kept in filtered and UV-radiated seawater (34 ‰ salinity), 12 °C (±1 °C) and on 24 h light. The fish were fed 1% of total biomass per day and starved for 24 h prior to handling and sampling. Before sampling, the fish were euthanized by bath immersion containing benzocaine chloride (1 g/5L water) (Apotekproduksjon AS, Oslo, Norway) for 5 min. The challenge trial was approved by the Norwegian Animal Research Authority and performed in accordance with the recommendations of the current animal welfare regulations: FOR-1996-01-15-23 (Norway).

### Experimental challenge

#### PRV challenge

**Figure 1:**
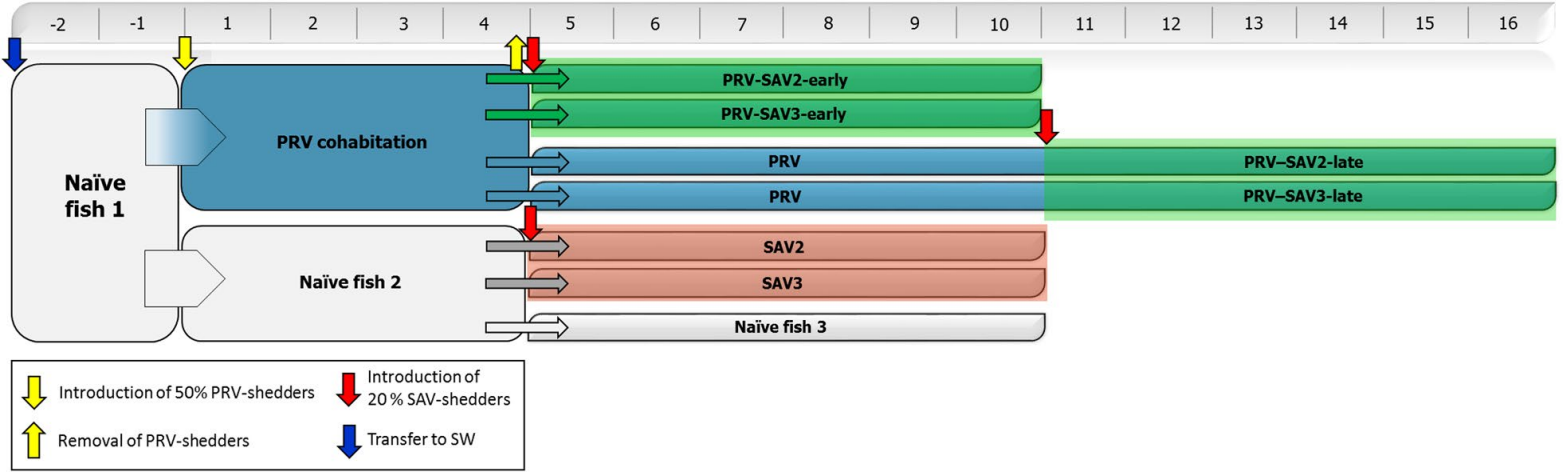
**The co-infection challenge trial.** The timeline above indicates weeks post PRV shedder introduction (WPC-PRV). Blue down arrow indicates transfer to sea water (SW). Yellow down arrow and yellow up arrow indicates introduction and removal, respectively, of PRV shedder fish. Red down arrow indicates introduction of SAV shedders. “Naïve fish 1” indicate the experimental fish before virus challenge, “Naïve fish 2” indicate fish dedicated to be SAV shedders and SAV controls at 4 WPC-PRV and “Naïve fish 3” indicate fish dedicated to be SAV shedders at 10 WPC-PRV. Blue box named “PRV cohabitation” indicates cohabitant fish exposed to 50% PRV shedders from 0 to 4 WPC-PRV. Blue box named “PRV” indicates PRV cohabitants from 4 to 10 WPC-PRV without PRV shedders. Boxes indicating a 6 week period of exposure to 20% SAV shedders are colored red (SAV only) and green (PRV-SAV). Co-infection induced at 4 WPC-PRV and 10 WPC-PRV is denoted PRV-SAV-early and PRV-SAV-late, respectively.

The inoculum (consisting of pelleted blood cells) was collected in a previous cohabitation trial (VESO Vikan), i.e. the second passage in experimental fish, and originated from a Norwegian field outbreak of HSMI in 2012. RTqPCR was performed as earlier described [34] and a high level of PRV RNA was indicated (Ct 17.3, using a total RNA input of 100 ng in the RTqPCR). The blood pellet was dissolved 1:1 in PBS and stored at −80 °C. On Day 0 of the trial, the blood pellet and PBS solution was thawed on ice, diluted 1:2 in PBS and 0.1 mL of the inoculum was i.p. injected into the anesthetized shedders. The inoculum was confirmed negative for IPNV, infectious salmon anemia virus (ISAV), SAV, piscine myocarditis virus (PMCV) by RTqPCR. After i.p. inoculation, the shedders (*N* = 363) were marked by adipose fin removal and placed in a tank containing naïve fish (*N* = 396). Four weeks post PRV shedder introduction (WPC-PRV), the PRV shedders were removed and the cohabitants were distributed into four tanks. As displayed in Figure 1, at 4 WPC-PRV, two tanks containing PRV cohabitants (*N* = 80) were supplied with either SAV2 (*N* = 20) or SAV3 (*N* = 20) shedders (1:4 ratio, shedder:cohabitant) starting the early co-infection. While the two other tanks contained PRV cohabitants until 10 WPC-PRV (indicated by “PRV” in Figure 1) and were sampled as PRV controls at 7 and 10 WPC-PRV. At 10 WPC-PRV, the PRV cohabitants (*N* = 80) in the tanks, were supplied with SAV2 (*N* = 20) or SAV3 (*N* = 20) shedders, initiating the late co-infection. Hence, cohabitants in the early and late co-infection were challenged with SAV shedders 6 and 12 weeks post sea water transfer, respectively.

#### SAV challenge

Naïve fish (*N* = 218) were kept in a separate tank (“Naïve fish 2”, Figure 1). SAV2 or SAV3 shedders were i.p. injected with 0.1 mL of cell culture medium containing SAV2 or SAV3 at a concentration of 10^4^ TCID_50_/mL. The SAV inocula were prepared as described earlier [21]. SAV shedders were marked by maxilla cutting. After injection, the SAV shedders were kept in separate tanks for four days before being introduced to the cohabitants. SAV2 or SAV3 shedders (*N* = 13) were placed in tanks with respective SAV subtype control tanks (*N* = 52 in each tank) (Figure 1). Naïve fish to be i.p. injected with SAV at 10 WPC-PRV were kept in a separate tank from 4 to 10 WPC-PRV (“Naïve fish 3” in Figure 1). Time after introduction of SAV shedders will be referred to as weeks post SAV shedder introduction (WPC-SAV). Organ samples from heart on RNA*later™* (Ambion Inc., USA) and heparinized blood were collected from naïve fish (*N* = 4) before SAV challenge. These were confirmed negative for SAV and PRV by RTqPCR. Due to differences in virulence and geographic distribution [21, 22], both SAV2 and SAV3 were included in the study.

#### Sampling

Eight cohabitants were sampled from the PRV only and PRV-SAV co-infected groups, whereas six cohabitants were sampled from the SAV control groups at each sampling. Eight fish sampled prior to PRV challenge served as negative controls. Weight and fork length was registered for all sampled cohabitants and Fulton’s condition factor (k-factor) was calculated (k-factor = weight in grams/length in cm^3^ × 100).

Tissue samples for histopathological evaluation (heart, pyloric caeca including exocrine pancreas and red and white skeletal muscle including the lateral line) were collected and fixed in 10% phosphate buffered formalin. After 24 h, the formalin was replaced with 70% ethanol and stored at 4 °C until further handling.

Two pieces of 2 mm^3^ from heart were collected on prefilled 1.0 mL tubes (FluidX^®^ Ltd, UK) with 0.5 mL RNA*later™* for RTqPCR analysis. Heparinized blood was collected from the caudal vein.

### Histopathology and immunohistochemistry

Samples for histopathology were processed and stained with hematoxylin and eosin following standard procedures. The sections from heart, pyloric caeca and skeletal muscle were examined blind and scored for pathological changes in an ordinal system (0, 1, 2, and 3), based on Taksdal et al. and McLoughlin et al. [21, 35]. The scoring performed in this study was modified and extended to include a separate score for acute myocardial necrosis and epicarditis as the former is a hallmark in PD development and the latter is observed in both PD and HSMI [9]. The scoring criteria for exocrine pancreas, myocardial degeneration and inflammation, acute myocardial necrosis, epicarditis and inflammation in skeletal muscle are displayed in Additional file 1.

Immunohistochemistry for detection of PRV and SAV in heart tissue were performed as described earlier for detection of PRV [13]; polyclonal rabbit anti-σ1 serum (1:2500) [13] for PRV and monoclonal murine anti-E2 (17H23) (1:2000) [36] for SAV were used as primary antibodies. Both were incubated overnight in a humidity chamber, SAV at room temperature and PRV at 4 °C. Biotinylated goat anti-rabbit (1:200) and biotinylated rabbit anti-mouse (1:300) were used as secondary antibodies (Dako, Agilent Technologies, Glostrop, Denmark). Vectastain ABC-AP kit (Vector, Laboratories, Burlingame, CA, USA) was used for visualization. Heart tissues from double infected fish tissues were investigated. As negative and positive controls, slides with SAV or PRV single infected tissues were included.

### RNA isolation and RTqPCR

All samples, including heparinized blood, were shipped cool (5–10 °C) and arrived within 24 h to the Norwegian Veterinary Institute laboratory after sampling. Tissue samples on RNA*later™* were placed at −20 °C until further analysis. A sub-sample of 200 µL from each heparinized blood sample was subsequently shipped cold, together with heart samples on RNA*later™*, to PatoGen AS for virus analysis.

PatoGen AS performed RNA extraction and RTqPCR analysis for PRV and SAV transcripts in heart and heparinized blood. The RTqPCR assay targeting PRV is validated to ISO17025 standards and was described by Glover et al. [37]. The SAV assay is validated and accredited to ISO17025 standards and was performed as described earlier [38]. Samples were defined as positive when having a PRV or SAV Ct lower than 37.0. Elongation factor 1α (EF1α) served as an internal reference gene [39] for all RTqPCR assays performed. PRV and SAV Ct values were normalized to EF1α Ct values (ΔCt = Ct^target^ − Ct^EF1α^). After finalizing the virus analyses, RNA (in RNase free H_2_O) extracted from heart and blood by PatoGen AS was shipped frozen on dry-ice, overnight to the NVI. RNA quantification and purity was determined using NanoDrop 2000 UV–Vis Spectrophotometer (Thermo Scientific, Wilmington, DE, USA). Finally, 1 µL RNase Out (0.5 U/µL, Life technologies) was added and the RNA was stored at −80 °C until gene expression analysis.

For gene expression analysis, cDNA was produced from 600 ng total RNA using QuantiTect Reverse Transcription Kit (Qiagen) according to the manufacturer’s instructions. Quantitative PCR was performed using 15 ng (5 µL of 3 ng/µL) cDNA input per reaction using Maxima SYBR Green (Thermo Scientific) with 10 µM of both primers. Primers are listed in Table 1. The cycling conditions used were 95 °C for 10 min, then 40 cycles of 95 °C/15 s, 60 °C/30 s and 72 °C/30 s in a Mx3005P (Stratagene, La Jolla, CA, USA). A seven-point concentration grade standard curve (40–0.675 ng) was run during testing of the primer pairs.

**Table 1:** **Primers used for gene expression analyses**

Target name	Sequence	Amplicon length	Genbank no.
Calsequestrin	Fwd: ATCCAGATGACTTCCCGCTG Rev: CTGGGGAGAGCCTAGGTCAAT	72	NM_001141681.1
Matrix metalloproteinase 13	Fwd: AGTGTCCAGCACAAATGACCT Rev: CTCAACTGCTGATCCACTGGT	78	XM_014163130.1
Interleukin 1-receptor accessory protein-like 2	Fwd:CTGGCTGGTCAATGGGACAT Rev: GTGGACCTGAAGTCCTCTGC	144	XM_014137694.1
Neuropeptide Y1	Fwd: GCTACCCGGTCAAACCTGAA Rev: GGACTGTGGGAGCGTATCTG	194	XM_014178359.1
Serum amyloid A5 protein	Fwd: GGTGCTAAAGACATGTGGCG Rev: CCACTGGAACCCTGAACCAT	173	NM_001146565.1
Arginase 1	Fwd: TGGCGATGTGCCTTTGATTT Rev: ATCCCGCGGTTGTCCTTTT	208	NM_001141316.2
Arginase 2—mitochondrial	Fwd: AACACAGGGTTGTTGTCGGT Rev: AGAGTCGAAGCTGTTCCGTG	193	XM_014211724.1

### Microarray analyses

The analyses were carried out using NOFIMA’s Atlantic salmon oligonucleotide microarray SIQ-6 and bioinformatic package STARS [40]. The platform includes 15 k unique probes to protein encoding transcripts; the genes were annotated by functions (GO), pathways (KEGG) and custom vocabulary. Microarrays were manufactured by Agilent Technologies (Santa Clara, CA, USA) and unless indicated otherwise, the reagents and equipment were purchased from the same source. The microarray analyses were performed on RNA from heart tissue that was shipped overnight from NVI to NOFIMA on dry ice. RNA from uninfected hearts, sampled at Day 0, was used as a common reference in all hybridizations. RNA amplification and labelling were performed with a Two-Color Quick Amp Labelling Kit and a Gene Expression Hybridization kit was used for fragmentation of labelled RNA. Total RNA input for each reaction was 500 ng. After overnight hybridization in an oven (17 h, 65 °C, rotation speed 0.01 g), arrays were washed with Gene Expression Wash Buffers 1 and 2 and scanned with a GenePix 4100A (Molecular Devices, Sunnyvale, CA, USA). GenePix Pro 6.0 was used for spot to grid alignment, assessment of spot quality, feature extraction and quantification. Subsequent data analyses were performed with STARS. After filtration of low quality spots flagged by GenePix, Lowess normalization of log_2_-expression ratios (ER) was performed. Genes that passed the quality control in more than half of the samples were included in the subsequent analyses. Differential expression was assessed by criteria: ER > 1.75-fold and *p* < 0.05.

### Data analysis and statistics

The statistical analysis was performed using GraphPad Prism 7.0 (GraphPad Software inc., USA). Differences in viral RNA levels were calculated based on ΔCt values using the non-parametric Mann–Whitney unpaired rank test. Differences in gene transcript levels in heart tissue and histopathological scores between the groups were examined using the non-parametric Mann–Whitney unpaired rank test. An unpaired student t test was used to examine differences in k-factor and weight. Spearman’s rank correlation was calculated using STATA 13.1 (StataCorp, USA), for associations between viral RNA levels in SAV cohabitants and histopathology score of acute myocardial necrosis. In addition, association between gene expression (ΔCt) and histopathology score of both myocardial degeneration and inflammation and acute myocardial necrosis was calculated. In all calculations of differences, a *p* < 0.05 was considered statistically significant.

## Results

The cohabitant challenge experiment is displayed in Figure 1. Weeks post PRV shedder introduction are abbreviated as WPC-PRV and weeks post SAV shedder introduction as WPC-SAV. The challenge trial consisted of 4 or 10 weeks of PRV infection alone (WPC-PRV) and a subsequent 6-week PRV-SAV co-infection period. The different time-points for SAV shedder introduction, i.e. 4 and 10 WPC-PRV, are termed early and late co-infection, respectively. Hence, the terms used are PRV-SAV-early or PRV-SAV-late. The SAV control fish were challenged simultaneously with the early co-infection. The results originate from cohabitant fish unless specified otherwise.

### Mortality and growth

Mortality was low during the experiment. Three (0.76%) PRV infected fish died during the first 4 weeks, while one fish died in the PRV-SAV2-early group (1.25%) between 4 and 10 WPC-PRV. The accumulated mortality at 6 WPC-SAV was 2.0 and 5.7% in the SAV2 and SAV3 controls, respectively. In the late co-infection there were no mortalities.

On Day 0, the mean weight, length and condition (k)-factor was 105.6 g (range 70.6–160.4 g), 21.2 cm (range 18.5–24.5 cm) and 1.09 (range 1.02–1.17), respectively. At the time of SAV shedder introduction, the mean weight at 4 WPC-PRV was 114.7 g (*N* = 16, PRV early group) and 109.3 g (*N* = 4, naïve fish) and at 10 WPC-PRV, the mean weight was 157.4 g (*N* = 16, PRV late group). At 10 WPC-PRV, all groups had increased average weight and length. However, the mean k-factor was reduced in the PRV-SAV2-early (1.06), SAV2 (1.02) and SAV3 (1.02) groups and increased in PRV controls (1.10) and the PRV-SAV3-early group (1.11). The difference between the SAV control groups compared to the PRV controls and the PRV-SAV3-early group were significant (*p* < 0.05) at 10 WPC-PRV. At the end of the trial, i.e. 16 WPC-PRV, the PRV-SAV2-late and PRV-SAV3-late groups had a mean k-factor of 1.17 and 1.11, respectively. Additional file 2 shows detailed range of weight, length and k-factor.

### PRV infection kinetics

**Figure 2:**
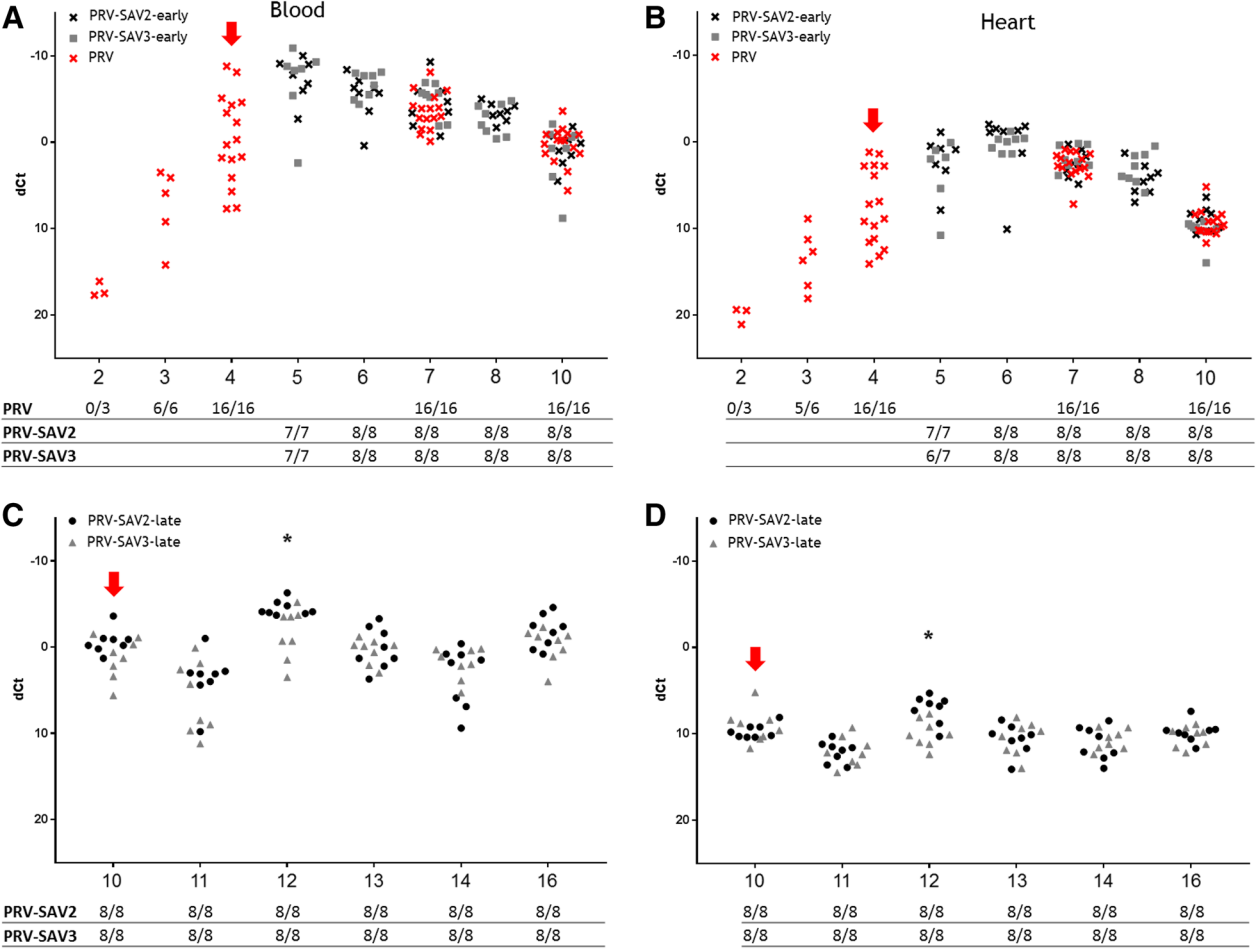
**PRV RNA kinetics.** PRV ΔCt values (PRV Ct–EF1α Ct) in blood and heart from individual co-challenged fish (black and grey dots) and PRV control fish (red x) assayed by RTqPCR targeting PRV RNA segments. Red arrow indicates introduction of SAV shedders early (**A**, **B**) or late (**C**, **D**). Weeks post PRV shedder introduction (WPC-PRV) are indicated at the x-axis. N positive individuals (raw Ct <37.0) of total sampled fish is tabulated under each sub-figure. Asterisk indicates significant (*p* < 0.05) differences in PRV RNA levels.

At day 0, the fish were confirmed negative for both PRV and SAV by RTqPCR. Successful transmission and infection of the cohabitants with PRV were confirmed by detection of viral RNA in blood and heart (Figures 2A–D). PRV was first detected in cohabitant fish at 3 WPC-PRV and the level of PRV RNA peaked at 5 WPC-PRV in blood (mean Ct 14.2) and 6 WPC-PRV in heart (mean Ct 18.3). High PRV RNA levels were present in the fish until the end of the experiment at 16 WPC-PRV (Figures 2C and D).

Histopathological changes including epicarditis and myocardial degeneration and inflammation in red skeletal muscle were in accordance to experimentally induced HSMI (Additional file 3), as previously described [13]. There were no differences in ΔCt values of PRV RNA or histopathological changes between the PRV controls and PRV-SAV groups. Likewise, there were no differences between the co-infected groups when comparing ΔCt values of PRV transcripts except a significantly lower ΔCt value in PRV-SAV3-late group compared to PRV-SAV2-late group at 12 WPC-PRV, *p* < 0.05 (Figures 2C and D).

### SAV infection kinetics

SAV2 and SAV3 shedders were confirmed SAV positive by RTqPCR in heart and blood. The histopathological changes were characteristic for PD, i.e. loss of exocrine pancreatic tissue, acute myocardial necrosis and myocarditis.

#### SAV challenge at 4 WPC-PRV (early co-infection)

In the PRV-SAV2-early group, the levels of SAV RNA were significantly lower in blood at 3, 4 and 6 WPC-SAV compared to the SAV2 controls (*p* < 0.05) and in heart at 4 and 6 WPC-SAV (*p* < 0.05). At both time points, 3/8 fish were SAV negative in heart in the co-infected group.

**Figure 3:**
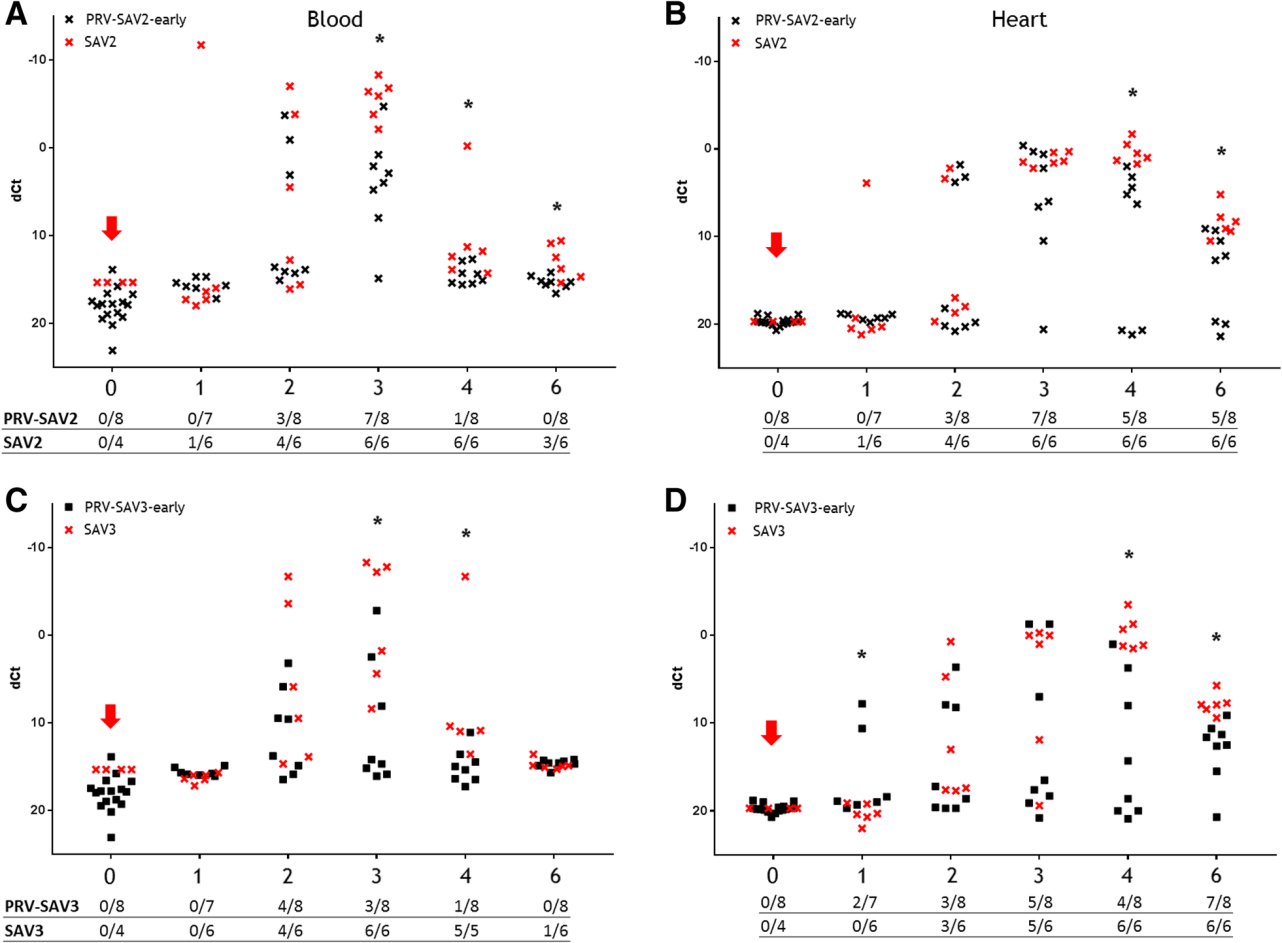
**SAV RNA kinetics, early co-infection.** SAV ΔCt values (SAV Ct–EF1α Ct) in blood and heart from individual co-challenged fish (black dots) and SAV control fish (red x) assayed by RTqPCR targeting SAV RNA segments. Red arrow indicates introduction of SAV2 shedders (**A**, **B**) or SAV3 shedders (**C**, **D**). Weeks post SAV shedder introduction (WPC-SAV) are indicated at the x-axis. N positive individuals (raw Ct < 37.0) of total sampled fish is tabulated under each sub-figure. Asterisk indicates significant (*p* < 0.05) differences between co-challenged groups and SAV control group.

The SAV RNA levels in the SAV3 control group peaked 3 WPC-SAV, however the PRV-SAV3-early group did not reach the same level (Figures 3C and D). At 3 and 4 WPC-SAV, the SAV RNA levels in blood was undetectable in more than 50% of the fish in the co-infected group and significantly lower than in the SAV3 control group (*p* < 0.05). The SAV RNA level in heart was significantly lower in the co-infected fish at 4 and 6 WPC-SAV compared to the SAV3 controls *(p* < 0.05).

#### SAV challenge at 10 WPC-PRV (late co-infection)

**Figure 4:**
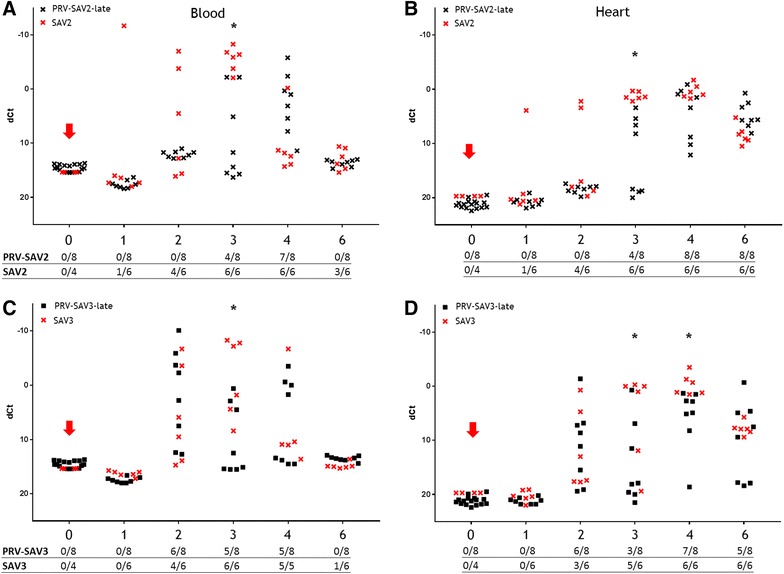
**SAV RNA kinetics, late co-infection.** SAV ΔCt values (SAV Ct–EF1α Ct) in blood and heart from individual co-challenged fish (black dots) assayed by RTqPCR targeting SAV RNA segments. For illustration, SAV ΔCt values from SAV control fish (red x) obtained during the early co-infection, was included in the figure. Red arrow indicate introduction of SAV2 shedders (**A**, **B**) or SAV3 shedders (**C**, **D**). Weeks post SAV shedder introduction (WPC-SAV) are indicated at the x-axis. N positive individuals (raw Ct < 37.0) of total sampled fish is tabulated under each sub-figure. Asterisk indicates significant (*p* < 0.05) differences between co-challenged groups and SAV control fish.

In the late co-infection, i.e. addition of SAV shedders at 10 WPC-PRV, SAV RNA was first detected in the PRV-SAV2-late group in blood and heart at 3 WPC-SAV and peaked at week 4 post SAV introduction, which was one week later than the control group (Figures 4A and B). The SAV RNA level in blood was significantly lower in the co-infected group at 3 WPC-SAV compared to the SAV control groups (*p* < 0.05). Furthermore, the co-infected group had a significantly lower SAV RNA level in heart compared to the controls at 3 WPC-SAV (*p* < 0.05).

In the PRV-SAV3-late group the SAV RNA levels did not show the same delay as observed for the PRV-SAV2-late group (Figures 4C and D). SAV RNA was first detected 2 WPC-SAV and reached peak levels 4 WPC-SAV in both heart and blood. However, at 3 and 4 WPC-SAV the SAV RNA level was significantly lower in heart in the co-infected group (*p* < 0.05). In blood, the SAV RNA level was significantly lower at 3 WPC-SAV in the PRV-SAV3-late group compared to the SAV3 controls (*p* < 0.05).

**Figure 5:**
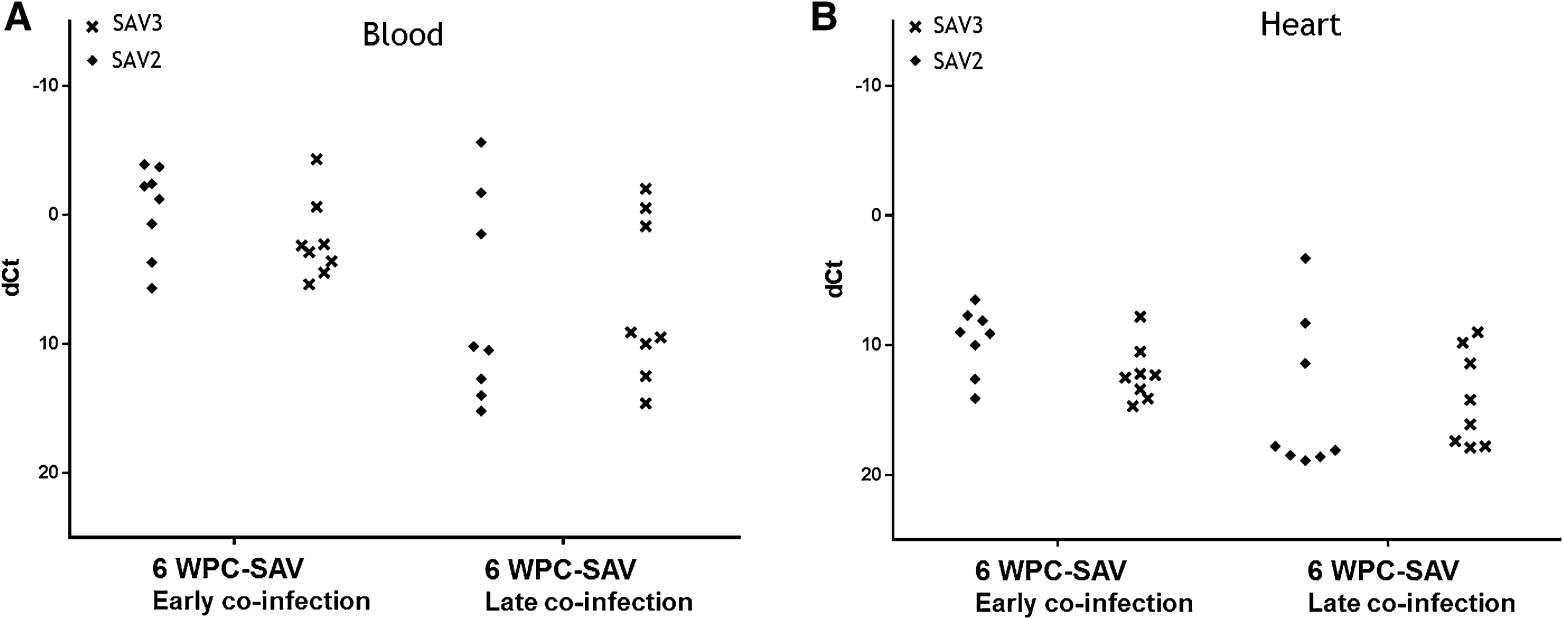
**PRV RNA levels in SAV2 and SAV3 shedder fish after 6** **weeks in the tank with PRV infected fish.** PRV ΔCt values (PRV Ct–EF1α Ct) assessed by RTqPCR targeting PRV RNA in blood (**A**) and heart (**B**). WPC-SAV indicates weeks post SAV2 or SAV3 shedder introduction.

During the co-infection, the SAV shedders also got infected with PRV (Figure 5). Although not significant, the PRV RNA levels in the SAV2 shedders after 6 WPC-SAV, were higher in heart when compared to SAV3 shedders.

### Histopathology in co-infected groups

**Figure 6:**
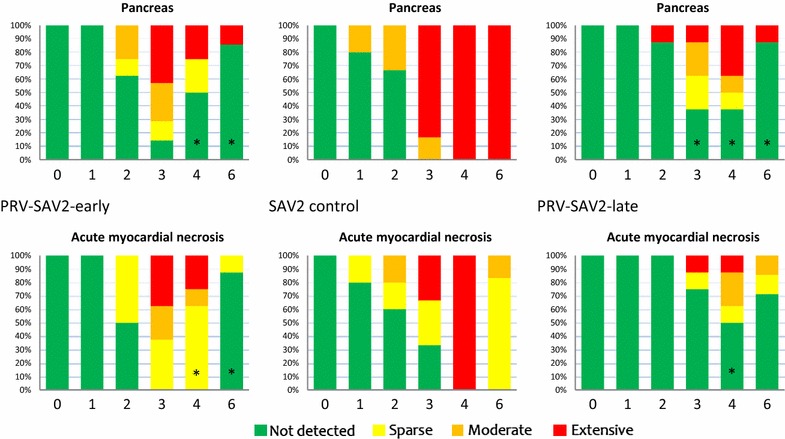
**Histopathological evaluation of PRV-SAV2 co-infected fish.** Histopathological scoring of PD associated changes in pancreas and acute necrosis in myocardium detected during the 6 week co-infection. Asterisk indicates significant (*p* < 0.05) differences between co-infected groups and SAV2 control fish. Week 0 represent uninfected control fish.

**Figure 7:**
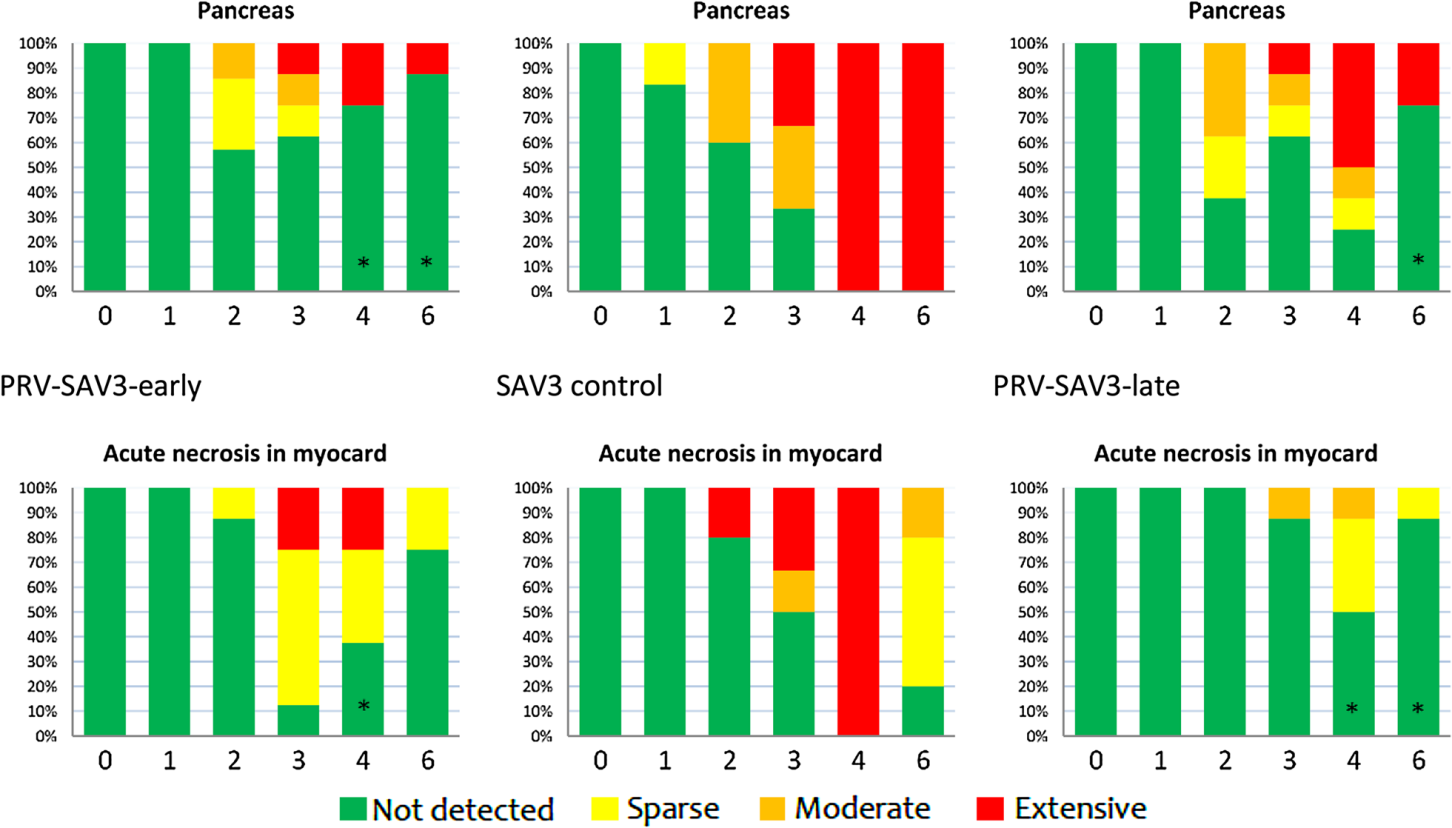
**Histopathological evaluation of PRV-SAV3 coinfected fish.** Histopathological scoring of PD associated changes in pancreas and acute necrosis in myocardium during the 6 week co-infection. Asterisk indicates significant (*p* < 0.05) differences between co-infected groups and SAV3 control fish. Week 0 represent uninfected control fish.

Histopathological scoring of changes in pancreas and acute myocardial necrosis showed a reduction in the co-infected groups compared with SAV control groups (Figures 6 and 7). Using a non-parametric rank test of the ordinal histopathological score, changes in pancreas were found to be significantly lower at 4 and 6 WPC-SAV in both early and late co-infection compared to the SAV control groups (*p* < 0.05), except at 4 WPC-SAV in the PRV-SAV3-late group (Figures 6 and 7). The PRV-SAV2-late group had also a significantly lower score in pancreas compared to the SAV2 control group at 3 WPC-SAV (*p* < 0.05). The prevalence of acute myocardial necrosis was significantly reduced at 4 WPC-SAV in the co-infected groups compared to the SAV controls (*p* < 0.05). At 6 WPC-SAV, the prevalence of acute myocardial necrosis was significantly lower in the PRV-SAV3-late and PRV-SAV2-early groups compared to the SAV controls (*p* < 0.05).

The SAV RNA levels (ΔCt) and histopathology score of acute myocardial necrosis showed a strong positive Spearman’s rank correlation (r_s_ = 0.81) in the SAV3 control group, *p* < 0.05 (*N* = 28), whereas a weaker correlation (r_s_ = 0.59) was seen in the SAV2 controls, *p* < 0.05 (*N* = 28).

**Figure 8:**
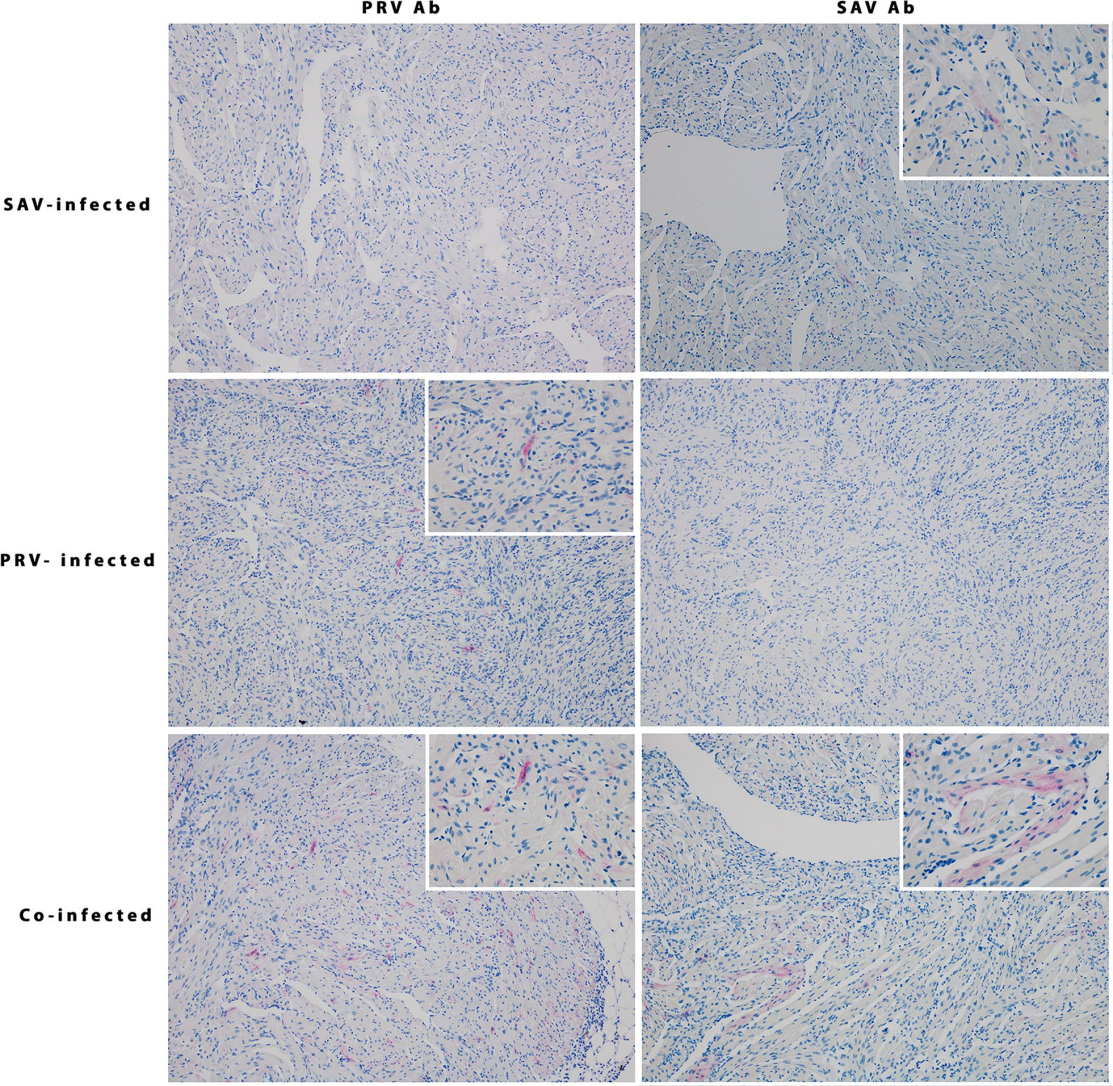
**Immunohistochemical detection of PRV and SAV in a co-infected heart.** Sections of heart tissue from SAV infected, PRV infected and PRV-SAV co-infected fish were stained using polyclonal rabbit antiserum targeting PRV σ1 (left panel) and monoclonal murine anti-SAV E2 (right panel). The fish were sampled at the same time point, 7 WPC-PRV which corresponds to 3WPC-SAV in the early co-infection. The small internal panels are enlargements to show specificity of the staining.

Immunohistochemistry (IHC) was performed on sections of heart tissue from single infected and co-infected fish. The fish were selected based on high viral levels (indicated by low Ct levels) of both viruses. The fish presented in Figure 8 were sampled 7 WPC-PRV, which for the SAV controls and co-infected groups correspond to three weeks post SAV introduction (3 WPC-SAV) in the early co-infection. Virus Ct values in the respective heart tissues are noted in Figure 8. In the SAV3 infected fish, sparse but distinct staining restricted to single cells was observed using SAV antibodies. The PRV antibodies yield a weak pink background color in both PRV infected and SAV infected heart tissues. However, distinct staining was only observed in PRV infected heart. Staining of both SAV and PRV antibodies were achieved in two separate sections of a co-infected heart. In the co-infected heart tissue more diffuse staining was observed in epicard, interpreted as unspecific binding for both SAV and PRV antibodies. When staining the control tissue with PRV antibodies, a weak pink background color was observed. Additional files 4, 5 and 6 include more detailed pictures. The IHC demonstrates the presence of PRV and SAV in the same areas of a co-infected heart section. However, no individual cells could be defined as co-infected.

### PRV-SAV co-infection and gene expression linked to heart pathology

**Figure 9:**
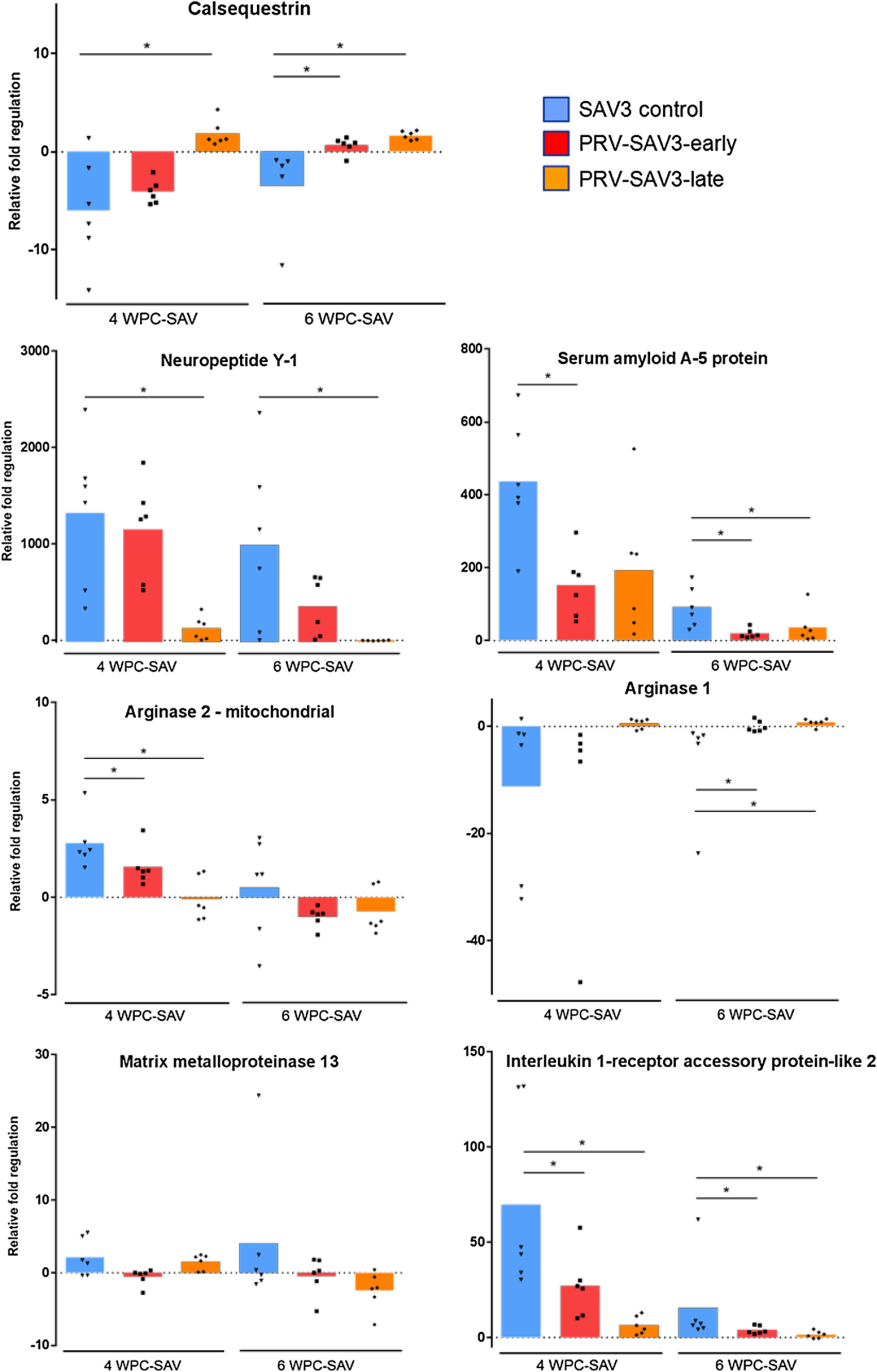
**Expression of potential pancreas disease marker genes in heart.** Fold induction or repression of genes identified as potential PD associated genes assessed in SAV3 infected (blue bars) and co-infected (red bars: PRV-SAV3-early, orange bars: PRV-SAV3-late) 4 or 6 weeks after SAV3 shedder introduction. Significant differences between SAV3 controls and co-infected groups are indicated with *p < 0.05. Boxes indicate mean fold change relative to mean levels at 4 WPC-PRV.

Microarray analysis was performed on hearts sampled at 4 and 6 WPC-SAV from the late co-infection and differences between the SAV3 control and PRV-SAV3-late group were analyzed (Table 2). Genes were selected based on their correlation with severity of pathological changes in heart induced by SAV infection, as previously reported [41]. To confirm the array results, RT-qPCR assays were run for seven selected genes, of which six showed significant differences (Figure 9). The gene regulation relative to 4 WPC-PRV (set to zero) is shown in Figure 9. At this time point the heart appeared healthy by histopathological evaluation. Gene expression at 4 and 6 WPC-SAV in both the early and the late co-infected group were compared to 4 and 6 WPC-SAV in the SAV3 control group. Significant differences were found between PRV-SAV3-late and SAV3 control for calsequestrin, neuropeptide Y-1 and interleukin 1-receptor accessory protein-like 2 (IL1R-2) at both 4 and 6 WPC-SAV, at 4 WPC-SAV for mitochondrial arginase-2 and at 6 WPC-SAV for arginase-1 and serum amyloid A5-protein (SAA5) (*p* < 0.05). The early co-infected groups differed significantly from the SAV controls for IL1R-2, mitochondrial arginase-2 and SAA5 at 4 WPC-SAV, and for calsequestrin, IL1R-2, arginase-1 and SAA5 at 6 WPC-SAV (*p* < 0.05). No significant differences between the groups were found for matrix metalloproteinase 13 (MMP13) expression.

**Table 2:** **Fold expression levels relative to naïve fish at day 0 of selected genes from microarray analysis at 4 or 6 WPC-SAV in PRV-SAV3-late and SAV3 control groups**

Gene	PRV-SAV3-late	SAV3 control	Difference	*p* value
4 WPC-SAV				
Serum amyloid A-5 protein	2.79	4.15	−1.36	0.212
Interleukin 1-receptor accessory protein-like 2	1.29	4.26	−2.98	0.040
Matrix metalloproteinase 13	0.97	5.67	−4.69	0.015
Calsequestrin	0.62	−3.02	3.63	0.003
Neuropeptide Y-1	0.33	2.85	−2.53	0.002
Arginase-1	0.03	−1.95	1.98	0.001
Arginase-2, mitochondrial	0.80	2.60	−1.79	0.065
6 WPC-SAV				
Serum amyloid A-5 protein	1.67	4.98	−3.31	0.001
Interleukin 1-receptor accessory protein-like 2	0.36	3.89	−3.53	0.001
Matrix metalloproteinase 13	0.18	5.43	−5.25	0.001
Calsequestrin	0.29	−1.68	1.97	0.001
Neuropeptide Y-1	0.08	2.99	−2.91	0.001
Arginase-1	0.01	−1.51	1.52	0.001
Arginase-2, mitochondrial	0.11	3.17	−3.06	0.001

Histopathology scores (ordinal variable 0, 1, 2 and 3) of myocardial degeneration and inflammation and acute myocardial necrosis were correlated with gene expression levels from the qPCR, using Spearman’s rank correlation (Table 3). The groups investigated (*N* = 6/group) were PRV controls (4 and 10 WPC-PRV), PRV-SAV3-early and -late (4 and 6 WPC-SAV) and SAV3 controls (4 and 6 WPC-SAV), making a total of *N* = 46 fish evaluated. Significant correlation was found for all gene expression levels toward the score of acute necrosis and inflammation in myocardial tissue, except for MMP13 against inflammation (Table 3). When corrected for multiple comparisons by Bonferroni-adjusted significant level, IL1R2, neuropeptide Y-1, SAA5, mitochondrial arginase-2 against necrosis and calsequestrin, IL1R-2, neuropeptide Y-1, arginase-1 against inflammation remained significant.

**Table 3:** **Spearman’s rank correlation between gene expression (RTqPCR) and histopathology score of inflammation in myocardium and acute necrosis in myocardium**

Spearman’s rank correlation				
	Myocardial degeneration and inflammation		Acute myocardial necrosis	
	r_s_	*p* value	r_s_	*p* value
Matrix metalloproteinase 13	0.0301	0.8427	0.3573	0.0148
Calsequestrin	−0.5284	0.0002	−0.4372	0.0024
Interleukin 1-receptor accessory protein-like 2	0.527	0.0002	0.7391	<0.0001
Neuropeptide Y-1	0.7049	<0.0001	0.6288	<0.0001
Serum amyloid A-5 protein	0.341	0.0204	0.7386	<0.0001
Arginase-2, mitochondrial	0.3798	0.0092	0.7135	<0.0001
Arginase-1	−0.6536	<0.0001	−0.4278	0.0030

## Discussion

This study demonstrates that a primary PRV infection reduces disease development of a subsequent SAV infection with either SAV2 or SAV3, as evidenced by lower levels of SAV RNA, less severe PD pathological lesions and higher condition factors in the co-infected groups. The lack of parallel groups must be accounted for when evaluating the presented results. Nevertheless, the observation of a similar reduction in disease development for both SAV subtypes in co-infected fish strengthens the validity of the results.

The most pronounced evidence of protection was a reduction in PD specific pathological lesions in exocrine pancreas. Lesions in the pancreas are a hallmark of SAV infection and used diagnostically for separation of PD and HSMI [9]. The heart is a target organ for both PRV and SAV, with myocarditis and epicarditis observed in both diseases [8, 9, 13]. This may possibly mask the protective effect on SAV induced myocarditis and epicarditis in this study. Cardiomyocytic necrosis is a typical pathological finding of early stages of SAV infection [9], and is not considered a specific feature of HSMI. In our study we found that the early co-infected groups had a significantly lower degree of acute myocardial necrosis compared to SAV controls. A recent study indicate a possible difference in susceptibility to SAV infection depending on time following sea water transfer [42]. Therefore, we cannot exclude that the size of the fish and time after sea transfer may have an impact on the difference in protection observed between the SAV only control groups and the late co-infected groups in our study.

Co-detection of PRV and SAV in hearts have been shown by RTqPCR in farmed Atlantic salmon escapees in Norway [10]. Here, we demonstrate the presence of both viruses in neighboring cells in heart tissue by immunohistochemistry along with co-detection by RTqPCR. However, since the protective effect was observed in pancreas which is not a target organ for PRV, this indicates that the PRV mediated protection is due to systemic responses and not to interaction through co-infection in the same tissue. PRV utilizes erythrocytes for replication and dissemination in the fish [34], and the salmon erythrocytes can mount innate antiviral responses after PRV infection [12, 43].

The PRV control group had a significantly higher k-factor 10 WPC-PRV compared to the SAV control groups at the same time point. The k-factors in the SAV2 and SAV3 controls were lower compared to the corresponding double infected groups (PRV-SAV2-early and PRV-SAV3-early), although only significant for the PRV-SAV3-early group. The higher k-factor supports the observed less severe histopathological changes in the co-infected groups, confirming the reduced impact of SAV after a preceding PRV infection.

Our results are in accordance with previous co-infections in fish where reduced mortality was observed. Pre-exposure of rainbow trout with either the non-virulent cutthroat trout virus (CTV) (*Hepeviridae*), chum salmon reovirus (CSV), or IPNV, gave a four week protection to a subsequent IHNV infection [26–30, 44, 45]. A similar effect against ISAV, lasting eight weeks, was observed for IPNV infected fish. However in that study the ISAV challenge was given intraperitoneally [46]. This indicates that long-lasting cross-protection between non-related viruses in fish is a general phenomenon, although the duration of protection may vary. Our study suggest an inhibitory effect of PRV on a secondary SAV infection which may last for at least 10 weeks post PRV challenge, and thus a longer duration of protection compared to other interfering virus infections reported in salmonids [26, 46].

Activation of the antiviral innate immune response, where the type 1 interferon (IFN) system is central [47, 48], is a possible explanation for protection against unrelated viruses after a primary virus infection. Studies on SAV infection in cell cultures and in vivo have shown upregulation of IFNα and a number of interferon induced genes [49, 50]. However, these studies revealed a complex antiviral innate response after SAV infection leading to reduced SAV propagation. PRV infection of Atlantic salmon RBC also cause strong up-regulation of antiviral genes of the innate immune response [43]. Two recent studies show that Mx expression is upregulated in heart tissue for 11 weeks [41], and in erythrocytes for at least eight weeks after PRV infection [43]. Interferon type I production was induced at the transcriptional level in erythrocytes for up to 7 weeks [43]. This suggests that circulating PRV infected erythrocytes could play an important role in the observed suppressive effect on SAV propagation and PD development by inducing interferon-regulated antiviral responses in most organs prior to SAV infection [51].

Therefore, at the time of early SAV shedder introduction in our trial, i.e. at 4 WPC-PRV, an upregulation of innate antiviral genes is expected. A possible long lasting innate immune response may contribute to the protection seen during the late co-infection. SAV RNA levels in heart were significantly lower 6 WPC-SAV in the early co-infected groups compared to the SAV controls. This difference was not present at the same time point in the late co-infected hearts, which could indicate a decreased protection caused by a reduction of innate immunity. However, since fish IgM have lower specificity and antigen affinity compared to mammalian serum antibodies, at least up to 15 weeks post infection, a mechanism of low affinity polyreactive natural antibodies cannot be ruled out [52, 53].

A possible difference was observed between the two SAV subtypes in the ability to handle the consequences of the preceding PRV infection. The SAV2 and SAV3 RNA levels differed in the co-infected groups. SAV2 replicated more efficiently than SAV3 during the early co-infection, whereas the peak phase of both viruses was lost in the late co-infection with PRV, either by a delay (SAV2) or by a reduction (SAV3). There were large individual variations in levels of SAV RNA and in prevalence of SAV positive fish in the co-infected groups, which makes it difficult to conclude if the apparent differences in SAV2 and SAV3 kinetics show true different properties of the virus subtypes.

SAV RNA kinetics in the heart tissue of both SAV2 and SAV3 controls were similar when assessed by RTqPCR, which is in accordance with a previous SAV challenge trial where several isolates were tested [21]. An interesting finding, that should be addressed in a suitable study, was the strong correlation between acute myocardial necrosis and SAV3 RNA level (r_s_ = 0.81) compared to SAV2 (r_s_ = 0.59) in the individual infected fish. If SAV3 is a stronger inducer of acute myocardial necrosis, this may partly explain the observed higher mortality associated with SAV3 compared to SAV2 [21, 22, 54].

A previous study using salmon microarray and RTqPCR analysis have reported that changes in the expression levels of certain genes are specifically associated with SAV mediated pathological changes in heart [41]. We found a similar regulation of these genes in the SAV controls in our study and that the expression levels of these genes were less affected in the co-challenge groups. This is in tune with the protective effects of a primary PRV infection, supporting histopathological observations and virus kinetics. RTqPCR run on a selected number of genes confirmed the microarray results. In general, the gene expression pattern was more affected in the SAV controls compared to the co-infected group. A previous study indicated a difference in gene expression patterns between the two diseases [41]. Our study found that the expression differences between the co-infected and SAV3 control groups changed in line with score of histopathological lesions, with a strong correlation of neuropeptide Y-1 and arginase-1 expression to the score of myocardial degeneration and inflammation. In mammals, neuropeptide Y has been shown to have several effects on inflammatory responses and cardiomyopathy [55, 56]. Furthermore, expression of IL1R-2, SAA5 and mitochondrial arginase-2 show a strong and significant correlation towards acute myocardial necrosis. Thus, genetic analysis may prove to be an additional tool for evaluation of the severity of salmon heart disease and tissue damage.

A peculiar finding was the secondary PRV infection detected in the SAV shedders who resided with the PRV cohabitants. This was observed in both the early and late co-infection. During the early co-infection, SAV2 shedders had a higher PRV RNA level, although not significant, in heart when compared to SAV3 shedders six weeks after introduction to PRV cohabitants. This may suggest that SAV3 yields a stronger protection against PRV than SAV2. A possible explanation is that SAV3, reported by others to be more virulent [21, 22, 54], yield a stronger immune response than SAV2. However, the lack of parallel tanks and number of fish per group (*N* = 6) must be accounted for when interpreting these results. The strong correlation between SAV RNA levels and myocardial necrosis may be a novel step towards understanding the observed virulence differences between the subtypes. An interesting observation in this perspective is the higher prevalence of HSMI in the geographically separated endemic areas of SAV2 in mid-Norway compared to those of SAV3 further south [4, 7]. These field observations could potentially be linked to a higher possibility of cross-infection between SAV2 and PRV and more prevalent development of both diseases in PRV-SAV2 dual infected fish.

Interactions between viral diseases may be part of the explanation for the large variation in severity described for SAV infections in the field [24]. PRV is found to infect fish in fresh water facilities and is ubiquitous in sea farms [6]. The protective effect in this study could affect the outcome of a PRV-SAV co-infection after sea transfer. In this study, the experimental fish had high levels of PRV RNA and developed HSMI. A more subtle PRV infection, where there is no HSMI development may cause a difference in strength and duration of the protection. Further research should address various field conditions when assessing the implications of PRV-SAV co-infection.

In conclusion, we found that a primary PRV infection partially protects against the outcomes of SAV infection and PD pathological lesions.

## Additional files

**Additional file 1.** **Scoring criteria for histopathological changes.** The table explains the categories of inflammatory changes that was used in the histopathological scoring.

**Additional file 2.** **Mean weight and k-factor is presented with range in brackets.** Accumulated mortality is presented in N with% in brackets. Number indicates weeks post PRV challenge. PRV-SAV2/3e and PRV-SAV2/3l indicates early and late co-infection, respectively.

**Additional file 3.** **Histopathology evaluation of epicarditis, pancreas pathology, acute necrosis in myocardium, inflammation in myocardium, inflammation in white and red skeletal muscle in PRV cohabitants.** Weeks after PRV challenge is shown. Week 0 represents uninfected control fish.

**Additional file 4.** **Immunohistochemistry of co-infected heart tissue stained using polyclonal rabbit antiserum targeting PRV σ1 (right panel) and monoclonal murine anti-SAV E2 (left panel).** The heart tissue had Ct values of 14.2 and 15.9 for SAV and PRV, respectively.

**Additional file 5.** **Immunohistochemistry of SAV-infected heart tissue stained using polyclonal rabbit antiserum targeting PRV σ1 (right panel) and monoclonal murine anti-SAV E2 (left panel).** The heart tissue had a SAV Ct-value of 17.1.

**Additional file 6.** **Immunohistochemistry of PRV infected heart tissue stained using polyclonal rabbit antiserum targeting PRV σ1 (right panel) and monoclonal murine anti-SAV E2 (left panel).** The heart tissue had a PRV Ct- value of 17.7.
