# Supplementary material for: Experimental Piscine orthoreovirus infection mediates protection against pancreas disease in Atlantic salmon (Salmo salar)
Source: Vet Res. 2016 Oct 21;47:107. doi: 10.1186/s13567-016-0389-y (PMC5075195; doi:10.1186/s13567-016-0389-y)
Supplement: Supplementary file 1 — Additional file 1. Scoring criteria for histopathological changes. The table explains the categories of inflammatory changes that was used in the histopathological scoring. [file 13567_2016_389_MOESM1_ESM.pdf]

Score	Criteria
Exocrine pancreas	
0	No pathological changes observed
1	Necrosis of exocrine pancreas, affecting up to 50% of exocrine tissues in the section
2	Necrosis of exocrine tissue affecting more than 50% of exocrine tissue in the section.
3	Absent of exocrine pancreatic tissue
Myocardial degeneration and inflammation	
0	No pathological changes observed
1	Focal myocardial degeneration and inflammation
2	Multifocal myocardial degeneration and inflammation
3	Extensive myocardial degeneration and inflammation (panmyocarditis)
Acute myocardial necrosis	
0	No pathological changes observed
1	Observed acute myocardial necrosis <10
2	Sporadic acute myocardial necrosis 10-50
3	Massive acute myocardial necrosis >50
Epicarditis	
0	No pathological changes observed
1	Sparse infiltration of inflammation cells in epicard
2	Moderate infiltration of inflammation cells in epicard, <3 layers
3	Extensive infiltration of inflammation cells in epicard >3 layers
Muscle lesion	
0	No pathological changes observed
1	Focal myocytic degeneration and inflammation
2	Multifocal myoctic degeneration and inflammation
3	Extensive myocytic degeneration and inflammation
