# Supplementary material for: Experimental Piscine orthoreovirus infection mediates protection against pancreas disease in Atlantic salmon (Salmo salar)
Source: Vet Res. 2016 Oct 21;47:107. doi: 10.1186/s13567-016-0389-y (PMC5075195; doi:10.1186/s13567-016-0389-y)
Supplement: Supplementary file 2 — Additional file 2. Mean weight and k-factor is presented with range in brackets. Accumulated mortality is presented in N with% in brackets. Number indicates weeks post PRV challenge. PRV-SAV2/3e and PRV-SAV2/3l indicates early and late co-infection, respectively. [file 13567_2016_389_MOESM2_ESM.pdf]

	Week 0				Week 4				Week 7			Week 10				Week 16			
	Mortality	Weight	K-factor	N	Mortality	Weight	K-factor	N	Weight	K-factor	N	Mortality	Weight	K-factor	N	Mortality	Weight	K-factor	N
PRV	0	105,6 (70,6-160,4)	1,094 (1,016-1,169)	8	3 (0,76)	114,7 (80,9-163,8)	1,051 (0,967-1,132)	16	148,3 (109,2-201,1)	1,099 (1,021-1,152)	16		157,4 (74,8-210,7)	1,103 (0,868-1,204)	16				
PRV-SAV2e									130,6 (116,8-150,7)	1,055 (0,906-1,175)	8	1 (1,25)	129,7 (76,1-201,4)	1,056 (0,813-1,196)	8				
PRV-SAV3e									131,1 (107,5-166,2)	1,115 (1,058-1,180)	8	0	171,1 (87,2-254,6)	1,106 (1,005-1,224)	8				
SAV2					0	109,3 (82,2-135,7)	1,087 (1,028-1,115)	4	159,6 (140,6-177,0)	1,063 (0,985-1,133)	6	1 (2,0)	128,5 (89,7-175,4)	1,015 (0,964-1,058)	6				
SAV3					0	109,3 (82,2-135,7)	1,087 (1,028-1,115)	4	151,7 (82,6-204,5)	1,077 (1,031-1,133)	6	3 (5,7)	141,1 (109,3-158,6)	1,016 (0,951-1,128)	6				
PRV-SAV2l												0				0	284,1 (237,5-348,4)	1,173 (1,099-1,256)	8
PRV-SAV3l												0				0	270 (65,4-343,4)	1,105 (0,882-1,264)	8
