# Supplementary figures and images for: Experimental Piscine orthoreovirus infection mediates protection against pancreas disease in Atlantic salmon (Salmo salar)
Source: Vet Res. 2016 Oct 21;47:107. doi: 10.1186/s13567-016-0389-y (PMC5075195; doi:10.1186/s13567-016-0389-y)

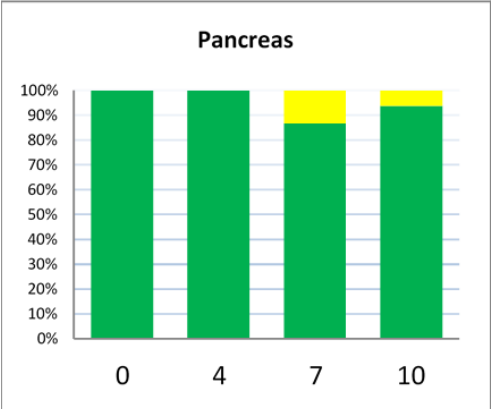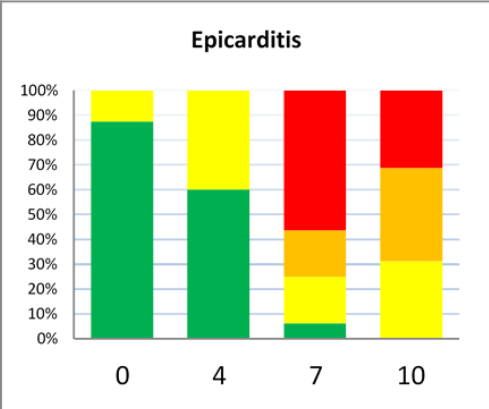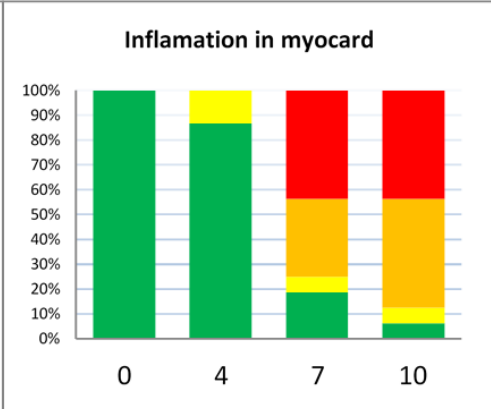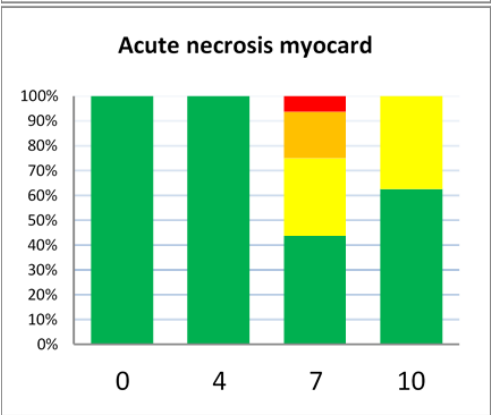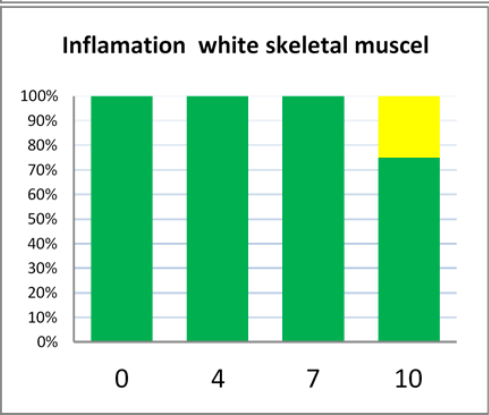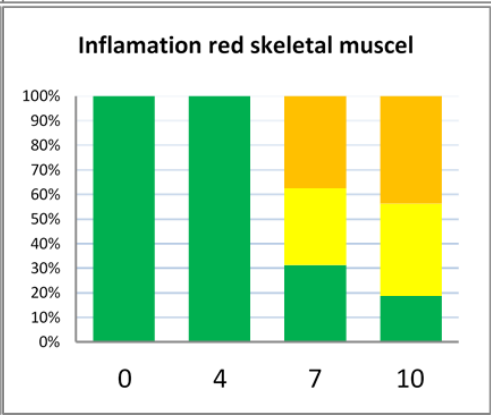

■ Not detected   ■ Sparse   ■ Moderate   ■ Extensive

Supplement: Supplementary file 3 — Additional file 3. Histopathology evaluation of epicarditis, pancreas pathology, acute necrosis in myocardium, inflammation in myocardium, inflammation in white and red skeletal muscle in PRV cohabitants. Weeks after PRV challenge is shown. Week 0 represents uninfected control fish. [file 13567_2016_389_MOESM3_ESM.pdf]

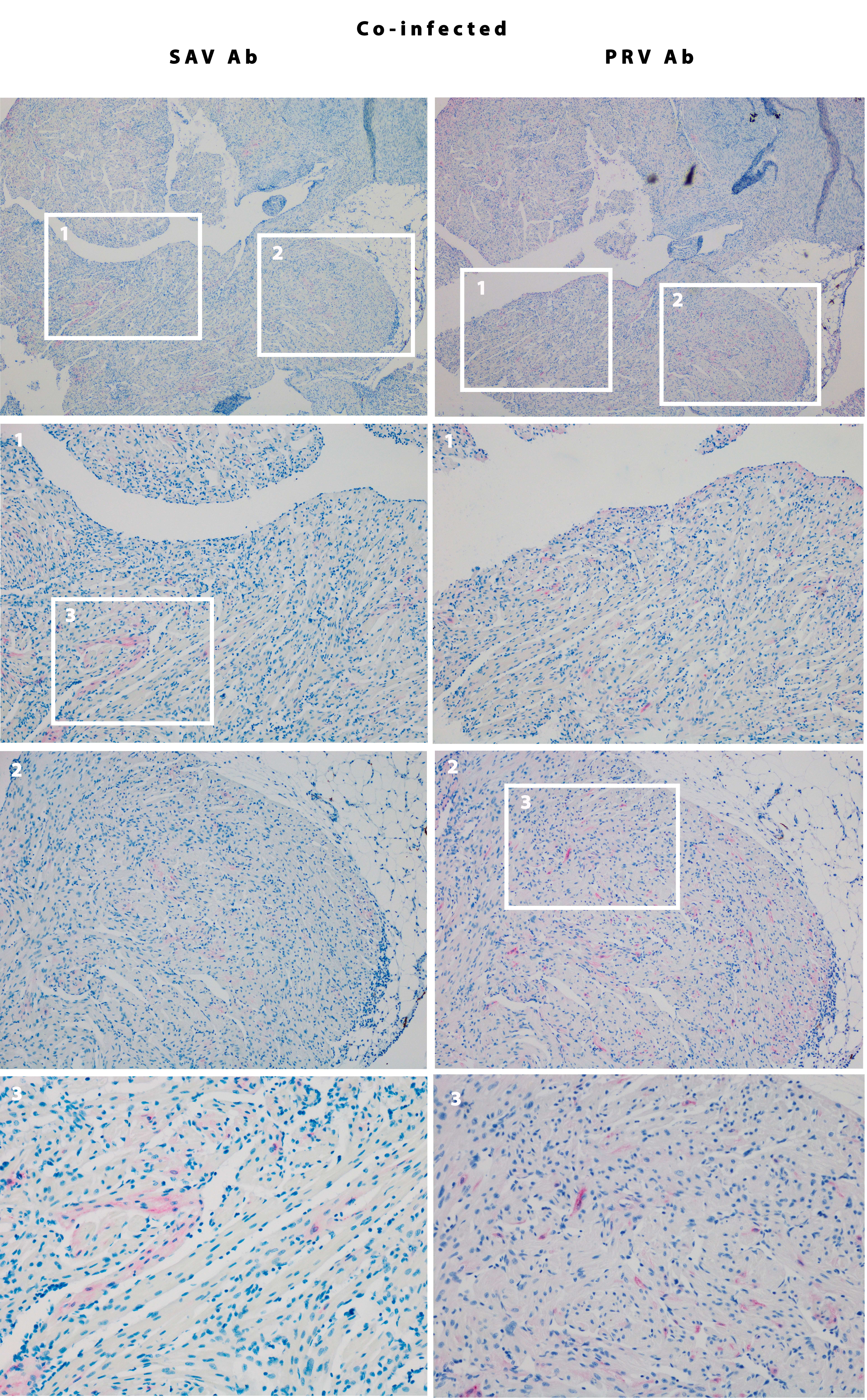

Supplement: Supplementary file 4 — Additional file 4. Immunohistochemistry of co-infected heart tissue stained using polyclonal rabbit antiserum targeting PRV σ1 (right panel) and monoclonal murine anti-SAV E2 (left panel). The heart tissue had Ct values of 14.2 and 15.9 for SAV and PRV, respectively. [file 13567_2016_389_MOESM4_ESM.jpg]

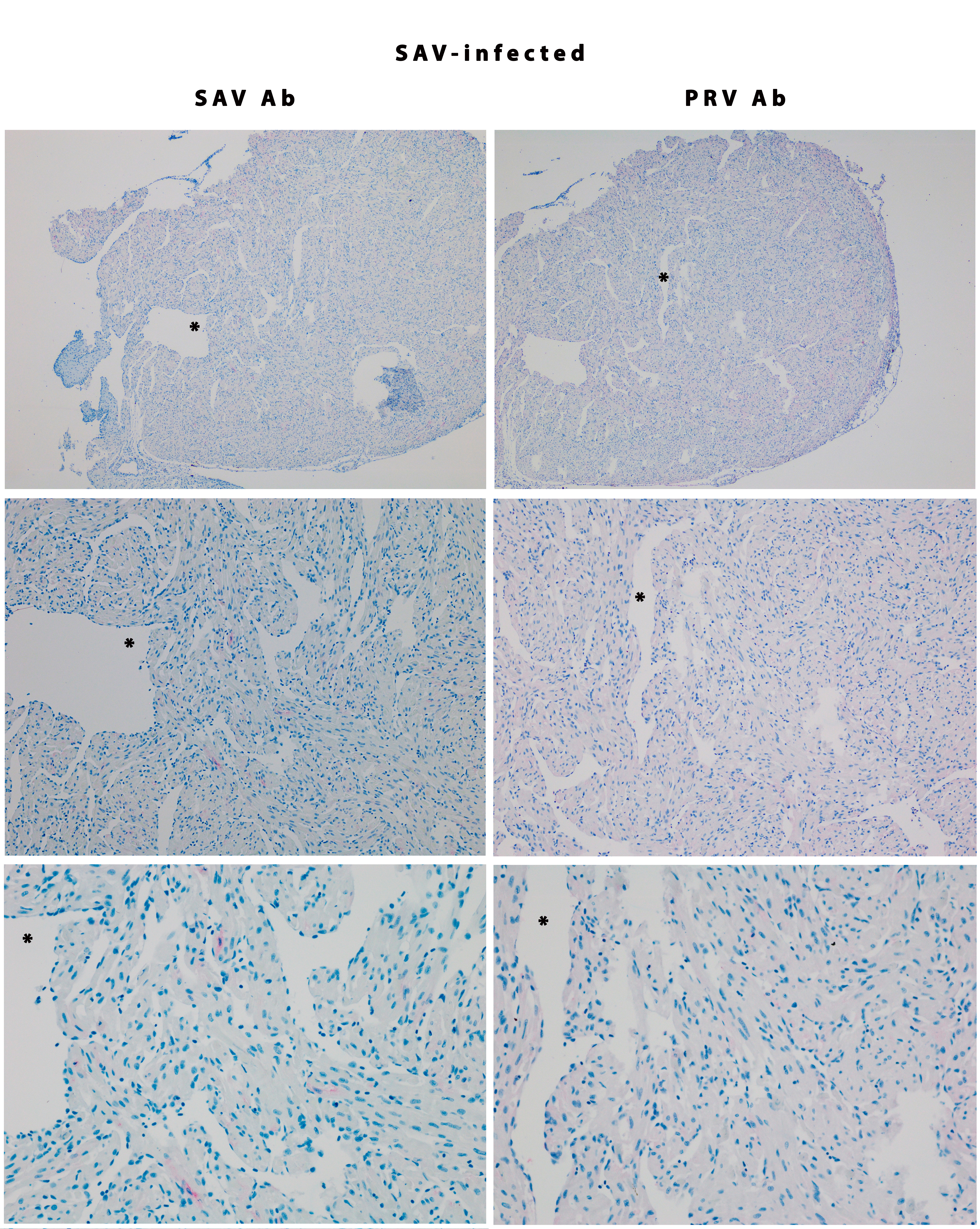

Supplement: Supplementary file 5 — Additional file 5. Immunohistochemistry of SAV-infected heart tissue stained using polyclonal rabbit antiserum targeting PRV σ1 (right panel) and monoclonal murine anti-SAV E2 (left panel). The heart tissue had a SAV Ct-value of 17.1. [file 13567_2016_389_MOESM5_ESM.jpg]

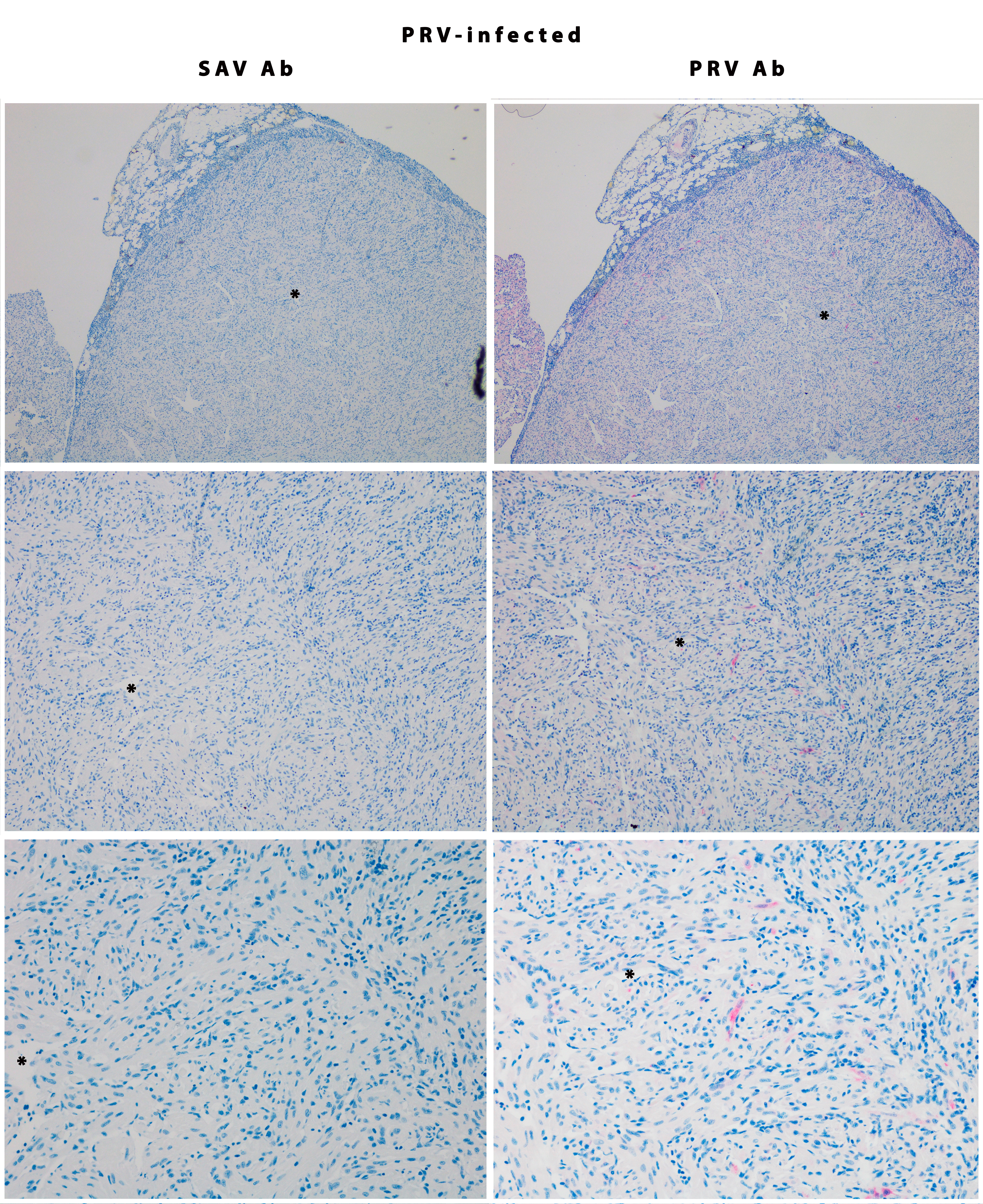

Supplement: Supplementary file 6 — Additional file 6. Immunohistochemistry of PRV infected heart tissue stained using polyclonal rabbit antiserum targeting PRV σ1 (right panel) and monoclonal murine anti-SAV E2 (left panel). The heart tissue had a PRV Ct- value of 17.7. [file 13567_2016_389_MOESM6_ESM.jpg]
